# The short-term associations of chronic obstructive pulmonary disease hospitalizations with meteorological factors and air pollutants in Southwest China: a time-series study

**DOI:** 10.1038/s41598-021-92380-z

**Published:** 2021-06-21

**Authors:** Meng Li, Shengqi Chen, Hanqing Zhao, Chengxiang Tang, Yunfeng Lai, Carolina Oi Lam Ung, Jinya Su, Hao Hu

**Affiliations:** 1 State Key Laboratory in Quality Research of Chinese Medicine, Institute of Chinese Medical Sciences, University of Macau, Macao SAR, China; 2 Sichuan Academy of Medical Sciences, Sichuan Provincial People’s Hospital, Chengdu, China; 3 School of Public Administration, Guangzhou University, Guangzhou, China; 4 School of Computer Science and Electronic Engineering, University of Essex, Colchester, CO4 3SQ UK

**Keywords:** Environmental impact, Risk factors, Respiratory tract diseases, Disease prevention, Public health

## Abstract

Chronic obstructive pulmonary disease (COPD) is the fourth major cause of mortality and morbidity worldwide and is projected to be the third by 2030. However, there is little evidence available on the associations of COPD hospitalizations with meteorological factors and air pollutants in developing countries/regions of Asia. In particular, no study has been done in western areas of China considering the nonlinear and lagged effects simultaneously. This study aims to evaluate the nonlinear and lagged associations of COPD hospitalizations with meteorological factors and air pollutants using time-series analysis. The modified associations by sex and age were also investigated. The distributed lag nonlinear model was used to establish the association of daily COPD hospitalizations of all 441 public hospitals in Chengdu, China from Jan/2015–Dec/2017 with the ambient meteorological factors and air pollutants. Model parameters were optimized based on quasi Akaike Information Criterion and model diagnostics was conducted by inspecting the deviance residuals. Subgroup analysis by sex and age was also performed. Temperature, relative humidity, wind and Carbon Monoxide (CO) have statistically significant and consistent associations with COPD hospitalizations. The cumulative relative risk (RR) was lowest at a temperature of 19℃ (relative humidity of 67%). Both extremely high and low temperature (and relative humidity) increase the cumulative RR. An increase of wind speed above 4 mph (an increase of CO above 1.44 mg/m^3^) significantly decreases (increases) the cumulative RR. Female populations were more sensitive to low temperature and high CO level; elderly (74+) populations are more sensitive to high relative humidity; younger populations (< = 74) are more susceptible to CO higher than 1.44 mg/m^3^. Therefore, people with COPD should avoid exposure to adverse environmental conditions of extreme temperatures and relative humidity, low wind speed and high CO level, especially for female and elderly patients who were more sensitive to extreme temperatures and relative humidity.

## Introduction

Chronic obstructive pulmonary disease (COPD) is a major chronic respiratory disease with high morbidity and mortality, threatening public health worldwide^1^. This disease is highly prevalent in the aging population and is projected to be the third leading cause of death by 2030^2^. Between 1990 and 2015, the global prevalence and death of COPD disease increased by 44.2% (17.5 million) and 11.6% (3.2 million). In China, the overall incidence of COPD in adults is about 8.6%, with people over 40 years old being as high as 13.7%. There were approximately 99.9 million COPD patients in China (as of 2018) and over 0.9 million people died prematurely because of COPD each year^3^, posing a substantial economic and social burden on patients and healthcare systems.

Tobacco smoking (active or passive) has been widely regarded as the most significant cause of COPD cases^4^. Besides smoking, it has been widely reported that exposure to noxious particles and gases are also important risk factors for COPD in previous epidemiological studies^5^. These adverse factors can reach the small airways and alveoli of the lung, promoting inflammation, and thereby exacerbating the underlying lung diseases and reducing the efficacy of lung-defense mechanisms^6^. There are also studies investigating the associations between COPD hospitalizations and ambient meteorological factors, since extreme meteorological factors potentially contribute to an increased risk of respiratory infection and decreased lung functions^7–9^.

It is noted, firstly, that most of the studies assessing the associations of COPD hospitalizations with environmental factors were carried out in Europe and the United States and little had been reported about Asian countries and regions. Apart from a small number of studies conducted in Hong Kong, Taiwan, and Beijing, the scarcity in the current understanding about the influences of environmental factors on COPD hospitalizations for patients in China was apparent. Secondly, the reported associations were inconsistent and varied across geographic locations, which was likely to be caused by the differences in environment, population demographics, and socioeconomic development^10^. Taking temperature as an example: some studies suggested that a decrease in ambient temperature was associated with a higher susceptibility to COPD risk^11^. However, a study in Brazil reported higher COPD risks during high temperatures^12^; while other studies suggested a reverse J-shape ^9^ or U-shaped association^10,13^. Regarding air pollutants^14^, a significant and positive association between COPD hospitalizations and Fine Particulate matter (PM2.5) was observed in Hong Kong^15^. However, a decrease of 3.9% in COPD hospitalizations per 15 μg/m^3^ increase in PM2.5 was observed in Birmingham, UK^16^ and no apparent association was found in a study in Rome^17^. Thirdly, very few studies considered the problem of nonlinear and lagged associations for COPD disease, which was commonly observed in related diseases such as asthma^10,17^, pneumonia^7^, lung function^8^, and tuberculosis^18^. Sex and age were also possible factors modifying the COPD-environment associations, since they might affect either physiological characteristics or health status of the COPD patients. Therefore, there is an urgent need to investigate the nonlinear and lagged associations of COPD disease with various environmental factors in an Asia area that considers the modified effects of sex and age.

Chengdu, located at the bottom of the Sichuan Basin, is the largest, most densely populated city and the economic and social center of Southwest China. With an elevation ranging from 450 to 720 m, Chengdu is surrounded by mountains and has a subtropical monsoon climate. Due to the special terrain characteristics, the dispersion of locally produced pollutants may be hampered, causing severe air pollution^18^. There are also more clouds and mist, less sunlight, with heavy humidity and mild wind speed^19^. These specific geographic and environmental characteristics make Chengdu a typical and representative city for assessing the effects of environmental factors on respiratory diseases, including COPD.

Understanding the associations of COPD hospitalizations with ambient environmental factors, and how the associations are modified by age and sex are paramount in devising health care guidance and policies, relieving the burden on patients and healthcare systems. Therefore, this study aims to evaluate the nonlinear and lagged associations of COPD hospitalizations with environmental factors in Chengdu by employing the distributed lag nonlinear model (DLNM)^20^. The modified associations by sex and age were also investigated via subgroup analysis. To the best of the authors’ knowledge, this is the first study to explore DLNM in investigating COPD disease for the large inland city in China that also considers the modified effects of sex and age.

## Results

### Basic variable characteristics

There were 397,026 COPD hospitalizations involving 245,363 males (61.8%), 151,451 females (38.1%) and 1180 missing (sex) data. The daily average male-to-female ratio, average patient age, and average COPD hospitalizations were 1.64, 73.6 years-old and 362 per day. The detailed statistical information on meteorological factors and air pollutants was summarized in Table 1.

**Table 1:** Descriptive statistics of COPD patient age, sex rate, daily hospitalizations, meteorological factors and air pollutants in Chengdu, China during Jan/2015–Dec/2017.

	Min	1st Qu	Median	Mean	3rd Qu	Max
Age	0	67	74	73.6	81	117
Male/female rate (daily)	0.93	1.50	1.64	1.64	1.78	2.64
COPD hospitalizations	27.0	285	344	362	436	1.17 × 10^3^
**Meteorological factors**						
Temperature (T: ℃)	1.72	11.6	19.1	18.4	24.7	32.5
Dew point temperature (DT: ℃)	9.44	7.39	14.1	13.5	19.6	25.8
Relative humidity (RH: %)	27.3	68.0	76.3	75.8	85.1	100
Wind speed (W: mph)	0.30	2.30	3.00	3.33	4.00	11.8
Atmospheric pressure (P: Hg)	27.7	28.0	28.2	28.2	28.4	29.0
**Air pollutants**						
PM2.5 (μg/m^3^)	0.0	32.0	49.0	60.1	77.0	313
PM10 (μg/m^3^)	0.0	55.0	82.0	99.1	126	126
SO2 (μg/m^3^)	5.00	10.0	13.0	13.7	17.0	38.0
CO (mg/m^3^)	0.50	0.80	1.00	1.08	1.20	2.80
NO_2_ (μg/m^3^)	15.0	41.0	50.5	52.4	63.0	121
O3 (μg/m^3^)	0.00	50.0	83.0	94.3	135	300

Min, Qu and Max mean minimum, quartile and maximum of the variables, respectively.

**Figure 1:**
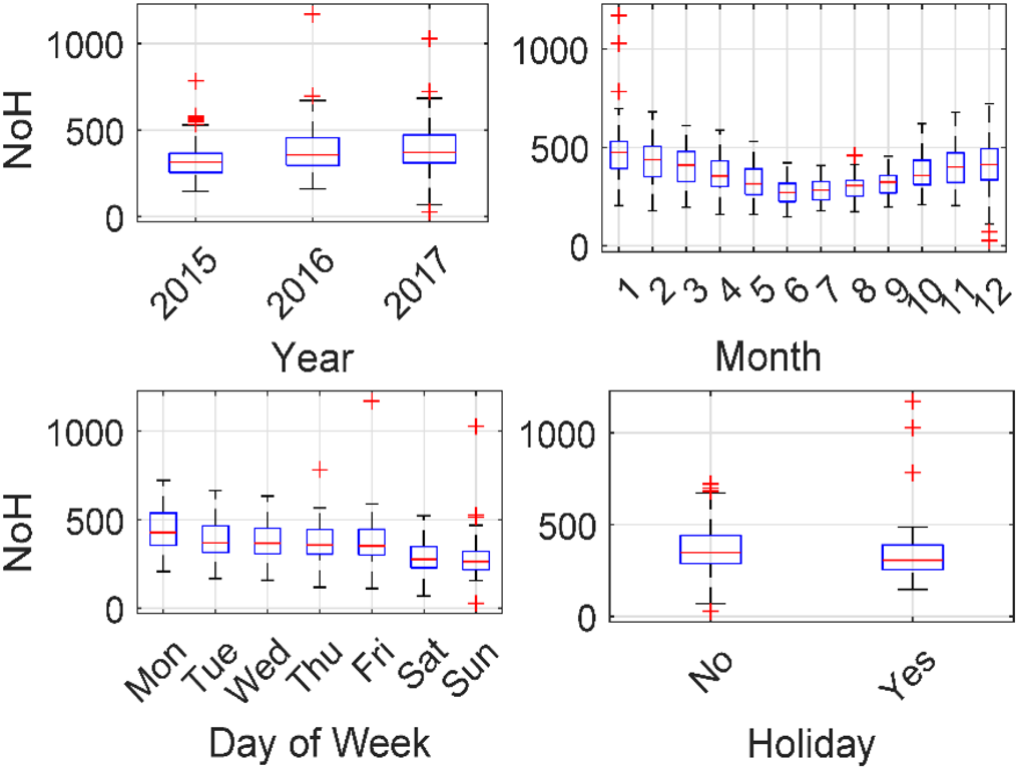
Boxplot of COPD hospitalizations against date related variables including year of study, month, day of week, and holiday.

The trend of the Number of COPD Hospitalizations (NoH) against year of study, month, Day of Week (DOW), and holiday is displayed in Fig. 1. It was shown that: (1) COPD hospitalizations increased in 2016 and 2017 as compared to 2015; (2) more COPD hospitalizations were recorded in cold season (November to April) compared to hot season (May to October); (3) fewer COPD hospitalizations were recorded on Saturday and Sunday compared to working days and most cases were hospitalized on Monday; (4) fewer COPD hospitalizations were reported during holidays.

COPD hospitalizations, meteorological factors and air pollutants over Day of Study (DOS) were displayed by stacked plot in Supplementary Fig. S1 online, where the time trend and rough associations could be observed accordingly. For instance, the trends between temperature and dew point temperature, PM2.5 and PM10 were very similar, implying a highly correlated relationship.

### Correlation analysis and variable selection

Spearman correlation coefficients for COPD hospitalizations, meteorological factors and air pollutants were shown in Supplementary Table S1 online, where all correlations were significant at the level of *P* < 0.01 except RH with PM2.5 (− 0.01*) and RH with NO2 (− 0.04*). Considering temperature was an important variable as suggested in the existing studies^20^ and^10^, dew point temperature and atmospheric pressure were excluded in the model due to their high correlations with temperature. In addition, the correlation coefficient between PM2.5 and PM10 was as high as 0.97, and therefore only one variable (PM2.5 without loss of generality) was kept in this study. For further investigation, meteorological factors including temperature, relative humidity, and wind, as well as air pollutants including PM2.5, SO2, CO, NO2, and O3 were analyzed.

### DLNM analysis

By choosing different model parameters such as maximum lag day (Mlag, Plag), Degree of Freedom (DOF) of nonlinear relationship (Mdof, Pdof) and the number of knots of lagged relationship (Mknots, Pnots) for environmental factors, 1008 models were compared for different groups respectively based on the qAIC value^20^. The optimized parameters for models of different groups are summarized in Table 2.

**Table 2:** Parameter optimization results based on qAIC value for different groups.

Model/parameter	Mlag [26:34]	Mdof [2, 3]	Mknots [2, 3]	Plag [6:12]	Pdof [2, 3]	Pknots [2, 3]
Whole population	31	3	3	10	2	2
Male group	30	3	3	11	2	2
Female group	31	3	3	10	3	2
Young group	31	3	3	11	3	2
Old group	31	2	3	12	2	2

#### Whole population analysis

**Figure 2:**
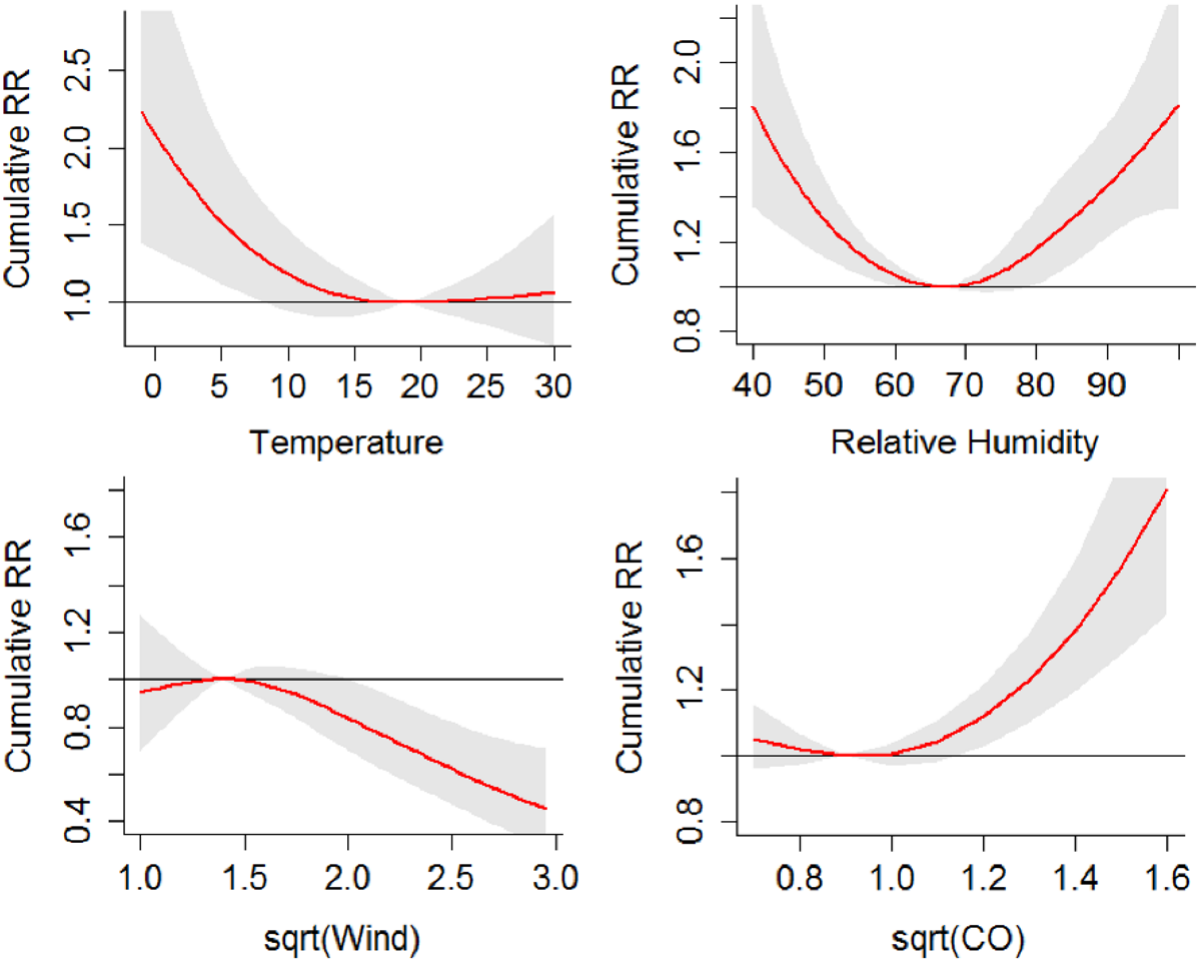
Plots of cumulative RR for COPD hospitalizations of the overall population (Jan/2015–Dec/2017, Chengdu, China) against: temperature, relative humidity, sqrt (wind speed) and sqrt (CO).

For the whole population, significant and consistent associations were observed for temperature, relative humidity, wind and CO. Their cumulative RR curves are shown in Fig. 2 depicting the following major observations. A hockey stick-shaped association was observed for daily mean temperature with the minimum value at 19 ℃. The cumulative RR increased as temperature increases (a little) or decreased from 19 ℃, where the association was significant for temperature less than 8 ℃ and insignificant for temperature over 20 ℃. A U-shaped association was observed for RH with the minimum RH at 67%. The cumulative RR increased as RH increased or decreased from 67%, with association being significant for RH less than 60% or over 79%. An inverted hockey stick-shaped association was observed for mean wind speed with the maximum value at 1.96 mph. The cumulative RR decreased insignificantly with value lower than 1.96 mph, and decreased with value higher than 1.96 mph where the association was significant for value over 4 mph. A hockey stick-shaped association was observed for CO level with the minimum value at 0.49 mg/m^3^. It appears that low CO level below 0.49 mg/m^3^ had certain (insignificant) protection effect and increase of CO above 1.44 mg/m^3^ resulted in a significant cumulative RR increase.

**Figure 3:**
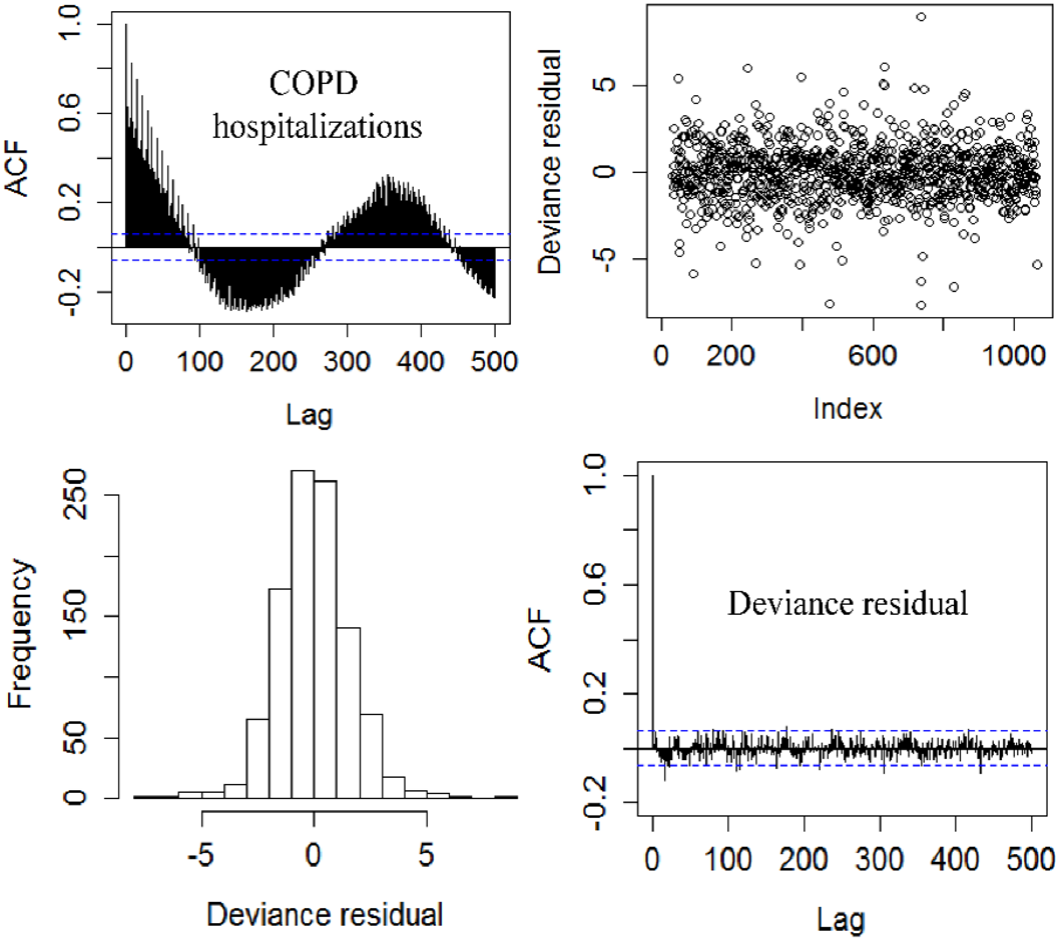
Results of model diagnostics: ACF values for COPD hospitalizations (top left), deviance residual against day of study (top right), histogram of deviance residual (bottom left) and ACF values for deviance residual (bottom right).

Model diagnostics was then conducted with the results in Fig. 3. Accordingly, there was an obvious time trend in ACF of COPD hospitalizations due to autocorrelation, however, the deviance residuals of the developed model were small (top right), nearly normal distributed (bottom left) and were generally within the 95% confidence level (bottom right), all indicating no obvious autocorrelation between deviance residuals. These results also confirmed the validity of the model.

#### Subgroup analysis by sex

**Figure 4:**
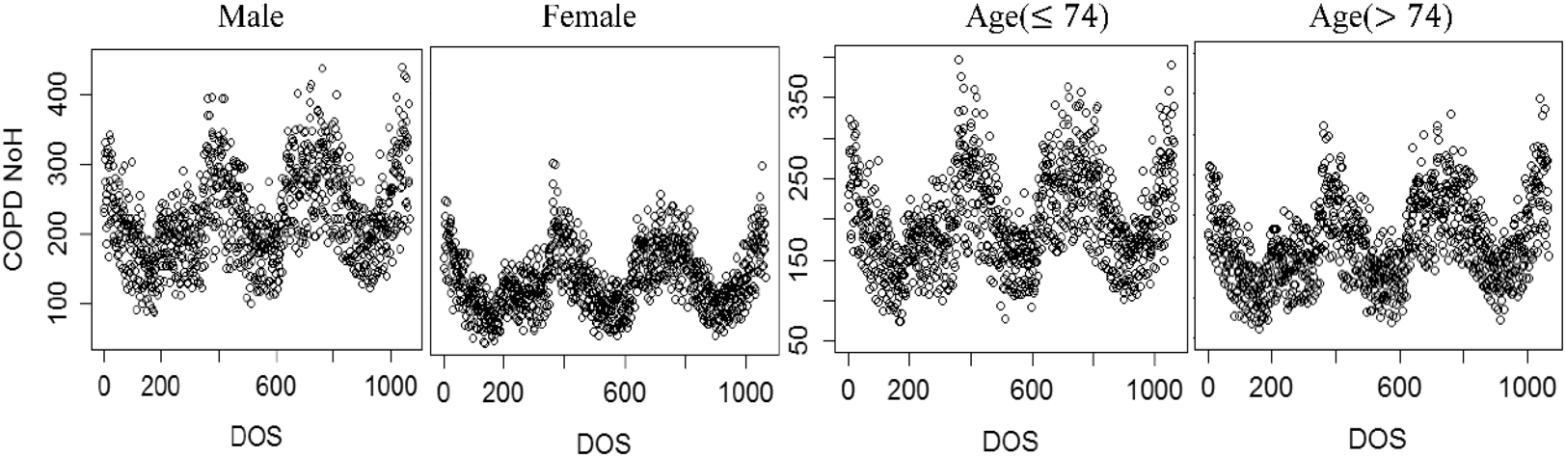
COPD daily hospitalizations categorized by sex (Male vs Female) and age (≤ 74 vs > 74) over the day of study (DOS).

**Figure 5:**
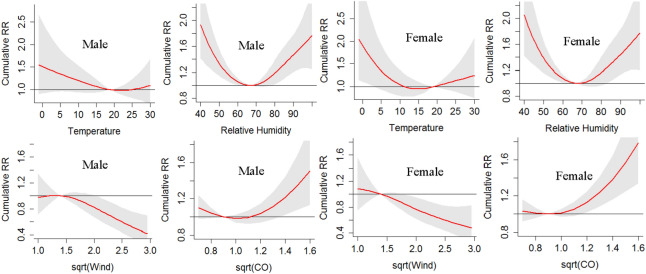
Plots of cumulative RR for Male (left) and Female (right) group: assessing the effect of sex.

Subgroup analysis was conducted for sex and age. COPD daily hospitalizations categorized by sex and age were displayed in Fig. 4, where one could see the differences among different subgroups. The results for Male group and Female group were displayed in Fig. 5. It can be seen that: (1) regarding temperature, female populations had a slightly lower value (15 °C vs 19 °C) of the minimum cumulative RR risk and were more sensitive to low temperature (significantly) and high temperature (insignificantly); (2) female populations were more sensitive to high CO level than male populations.

#### Subgroup analysis by age

**Figure 6:**
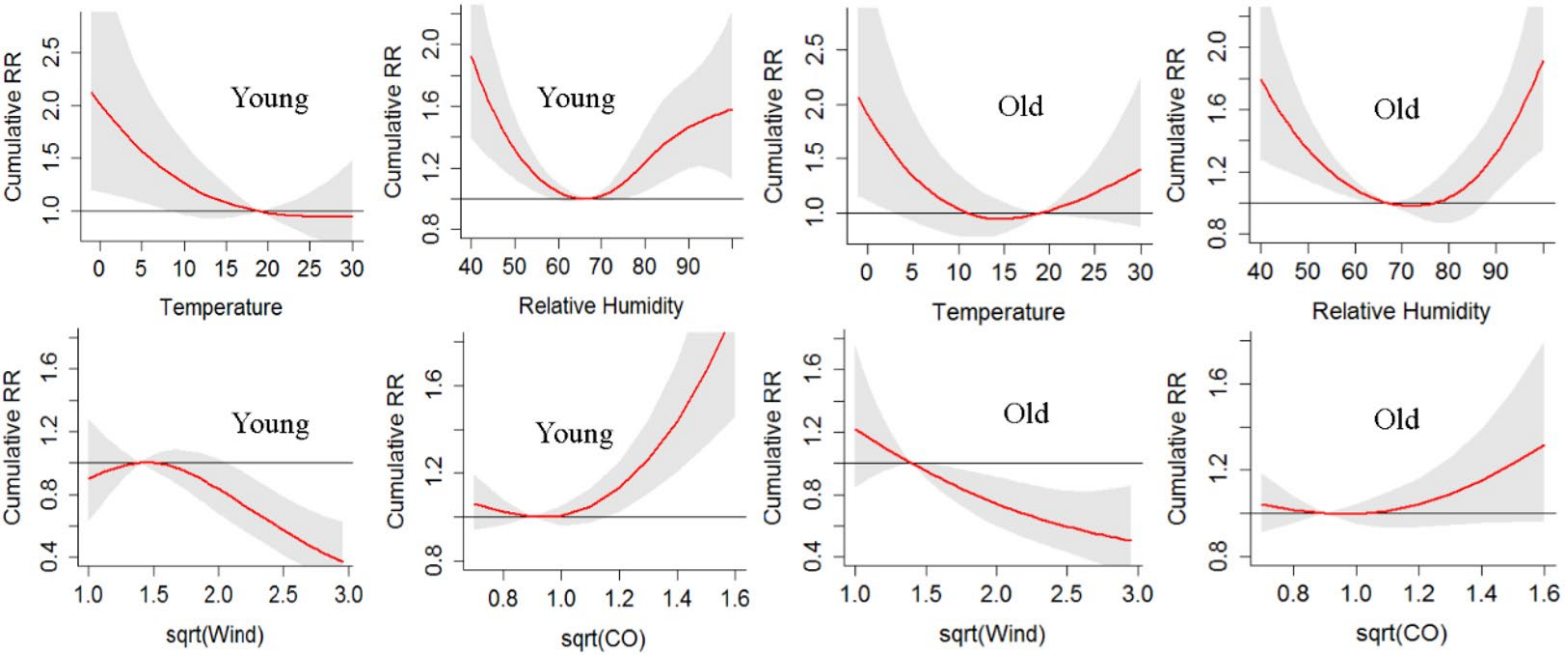
Plots of cumulative RR for the population with age $\le 74$ (left) and age > 74 (right): assessing the effect of age.

The age median value 74 was chosen as the threshold to divide the whole population into younger group ($\le 74$) and older group (> 74), where the corresponding associations were shown in Fig. 6. The following observations can be drawn: (1) the older population was more sensitive to high temperature, although high temperature was statistically insignificant for both groups; (2) regarding relative humidity, the older population had a slightly higher optimum value (74% vs 67%) of cumulative RR and were more sensitive to high relative humidity; (3) the younger population was more sensitive to high CO level while the association for CO level was insignificant for the older population.

## Discussion

This study shows that both extremely high and low temperatures are associated with an increased cumulative RR with extremely low temperature demonstrating a stronger and more significant association. This finding is consistent with the existing studies across different climatic conditions^10^. For example, a study in Korea^21^ and a cohort study in London^22^ showed that exposure to extremely low temperature was associated with more severer COPD exacerbations. There were also studies demonstrating an increase in hospitalization due to exacerbations on days with extremely high temperature^23^. For all the patients in this study, although the cumulative RR curve tended to increase at extremely high temperature, the association was not statistically significant. Similar observations were drawn in the studies conducted in Beijing ^10^ and London^24^. The underlying reason for the adverse effects of extreme temperatures was that extreme temperatures could increase the risk of respiratory infections and decreased lung functions^9,25,26^. The vulnerability to cold temperature than hot temperature exposure may be explained by the more access to air condition in hot weather^9^.

Regarding relative humidity, a U-shaped association was observed. Different from many existing studies in which RH was adjusted as a confounding factor^27^, its nonlinear and lagged effect was considered via cross basis function. This result is consistent with the existing studies. For instance, a hockey-stick association (with the minimum RH at 82%) was observed in Hong Kong for both pneumonia and COPD disease in cold seasons^7^. A study in Taiwan also showed that lower humidity was associated with an increase in COPD exacerbation^11^. Low humidity, together with low temperature, may induce bronchoconstriction, dry the mucosal membrane along the airway, and therefore increase susceptibility to airway bacterial and viral infections^10^, which altogether increase the risks of COPD hospitalizations. High RH also led to a higher relative risk of COPD hospitalizations. Because high RH usually resulted in high level of air pollutants, such as dust mites, bacteria and viruses, which may increase the risk of pollutant-induced triggers and worsen COPD symptoms.

Regarding wind speed, an inverted hockey stick-shaped association was observed. This may be explained by that Chengdu is located at the bottom of Sichuan Basin where low wind speed may hamper the dispersion of locally produced pollutants, leading to a higher cumulative RR. Therefore, it is speculated that relatively high wind speed has significant protection effects. It is noted, however, that the studies about the effects of wind speed on respiratory diseases are much sparser compared to other meteorological factors. One study in Bavaria, Germany showed that wind speed was only significant in the north due to the regional differences between north and south Bavaria^28^, highlighting the possible modified effect of terrain characteristics on wind speed. Another study in Hong Kong showed that high wind speed was associated with a lower relative risk of COPD^10^. Our results were not only aligned with the results in Hong Kong, but also suggested significant protection effects of high wind speed possibly due to the terrain characteristics of Chengdu.

An increase of CO level above certain threshold will significantly increase the cumulative RR, while low CO level has certain (insignificant) protection effect. The existing studies about the effects of CO on respiratory diseases are inconsistent across studies^29^, especially for CO at low concentrations. A positive association of CO exposure with emergency department visit for respiratory diseases was observed^30^, while a study in Shanghai shows that a negative association is found for low ambient CO concentration^14^. Our result, low CO level shows certain protection effect (insignificant), is aligned with the existing results and the result for high CO level again confirms its adverse effects on COPD hospitalizations.

Subgroup analysis by sex showed that female was more sensitive to extremely high (insignificantly), extremely low (significantly) temperature, and high CO level (significantly). These findings are consistent with the previous studies^9^, where the reason might be due to the biological susceptibility of female populations. The elderly (> 74) patients were more sensitive to extreme high temperature and high relative humidity, which could be due to their thermoregulation impairment, reduced immune function, higher likelihood of comorbidities and longer history of diseases^31^. Interestingly, younger patients (< = 74) were more susceptible to CO level. The results of age differences on the effects of air pollutants were inconsistent in previous studies^32,33^. Some studies show that older populations were more sensitive to air pollution due to their poor immune function^32^, while others confirmed that younger populations were more vulnerable due to a longer exposure time^33^. Our results are consistent with the latter. The possible reason is that CO is usually produced by the incomplete combustion of carbon-containing fuels and younger populations may have a higher likelihood of a longer exposure to CO due to their longer outdoor activities.

It is generally not easy to determine the lag days for meteorological factors and air pollutants. However, some existing studies have shown that a relatively longer lagged effect was observed for meteorological factors (e.g. 30 days for temperature) than air pollutants^9,10^. Therefore, on the basis of the existing studies, time ranges of 26–34 days and 6–12 days were selected for meteorological factors and air pollutants, respectively in this study, which were further optimized by using the qAIC value^20^. The optimized lag days for different gender and age groups were slightly different and the detailed information was available in Table 2.

The main strengths of this study are summarized. Firstly, this study involved a relatively large number of COPD hospitalizations from all 441 public hospitals in Chengdu. A large dataset can lead to a more reliable conclusion. Secondly, both nonlinear and lagged associations were considered by the state-of-the-art DLNM, which better reflected the real scenario as compared to the common nonlinear model without considering lagged effect. Thirdly, model parameter selection based on qAIC value was conducted for different population groups and model diagnostics was performed according to deviance residuals. The subgroup analysis by sex and age also helped draw more detailed observations.

A few limitations in this study were worth mentioning. For instance, this study assumed that the whole population had a same level of exposure to the environmental factors. Individual patient characteristics (e.g. smoking history, a history of prior hospitalizations and comorbidities), which might affect the associations, were not considered due to the lack of information. In addition, focused on COPD daily hospitalizations over meteorological and air pollution factors, this study did not consider the severity of COPD due to a lack of such information. It would be interesting to investigate the modified associations by COPD severity in our future research with their advent, which can be achieved by subgroup analysis. To summarize, individual patients’ characteristics (e.g. smoke history/current status, COPD disease severity) will be taken into account in our future research design, which will be treated as categorical factors at a micro (individual patient) level research (e.g., hospital length of stay, patient readmission risk assessment, hospitalization cost analysis) or as a grouping variable (in subgroup analysis) at a macro level research (e.g. daily hospitalization).

To conclude, people with COPD disease should avoid exposure to adverse environmental conditions by limiting their outdoor activities during periods of extreme temperatures (e.g. high or low), extreme relative humidity (e.g. high or low), low wind speed and high CO level. They are also encouraged to take various measures to keep the optimum indoor temperature and relative humidity to reduce the chance of hospitalization, especially for female and elderly patients. The results in this study can be taken into account in designing health precaution guidelines or policies for patients with COPD disease against extreme environmental conditions and can be referred in planning medical resources for healthcare systems in Chengdu or cities with similar geographic and environmental characteristics.

## Material and methods

### Data sources

Hospitalization information was collected from the Electronic Medical Record of all 441 public hospitals in Chengdu during Jan/2015–Dec/2017. According to the International Classification of Diseases code of version 10 (ICD-10), patient records with principal diagnosis at discharge of COPD (J44.0, J44.1, J44.8 and J44.9) were selected.

Meteorological data were collected from an international weather database (http://www.wunderground.com), which had been widely used in previous studies^34^. The averaged data from available weather stations in Chengdu were used, where the retrieved data included daily average temperature (T: ℃), dew point temperature (DT: ℃), relative humidity (RH: %), wind speed (W: mph) and atmospheric pressure (P: Hg).

Air pollutant data were collected from “China’s air quality online monitoring platform” https://www.aqistudy.cn/historydata/, which provided the average data from the available monitoring stations in the city and had been widely used in many studies in China^35^. The retrieved pollutant data included daily average fine suspended particles (FSP/PM2.5, μg/m^3^), average respirable suspended particles (RSP/PM10, μg/m^3^), average sulphur dioxide (SO2, μg/m^3^), average carbon monoxide (CO, mg/m^3^), average nitrogen dioxide (NO2, μg/m^3^), and maximum 8 h’ average ozone (O_3_ in μg/m3).

### Basic statistical analysis

Data visualization and Spearman correlation coefficient were first drawn to initially assess the correlation. Significance test was performed at a significant level of *P* < 0.01. It was noted that too many independent variables usually required a longer time-series dataset and highly correlated variables also impaired the model performance. Therefore, upon conducting the correlation analysis, some of the highly correlated variables were removed.

### DLNM

Upon choosing the predictors, the DLNM^20^, was then developed to describe the potentially nonlinear and lagged associations between COPD daily hospitalizations and environmental factors with the underlining model:

$$ \begin{aligned}   {\text{Log}}\left[ {{\text{E}}\left( {Y_{t} } \right)} \right] &  = cb\left( {Mfactors,~DOF = ;lag = ,~DOF = } \right) \\     & \quad  + cb\left( {Pollutants,~DOF = ;lag = ,~DOF = } \right) + ns\left( {DOS,~k = } \right) \\     & \quad  + factor\left( {Month} \right) + factor\left( {DOW} \right) + factor\left( {Holiday} \right) \\  \end{aligned} $$

On the left-hand side, $Y_{t}$, ${\text{E}}()$ and ${\text{Log}}()$ represented the daily COPD hospitalizations, expectation, and logarithm operation (link function for quasi-Poisson family). On the right-hand side, $cb\left( {x,~DOF = ;lag = ,~DOF = } \right)$ denoted the cross basis of the independent variable $x$ (e.g. meteorological factors $Mfactors$ or air pollutants $Pollutants$), which was available in the $dlnm()$ package in ${\text{R}}$ environment; $DOF =$ in cross basis denoted the degree of freedom, which was 2–5 for environmental exposures and 2–4 for lagged effect in similar studies^36^; $ns\left( {x,k = } \right)$ denoted the natural cubic spline smoothing function in R package ${\text{mgcv}}()$^37^, of the independent time variable $x$ with DOF $k$; $factor()$ represented the indicator of categorical independent variables. $DOS,{\text{~}}Month,~DOW$ and $Holiday$ denote the day of study (1,…,1096), month of year, day of week, and public holidays in China.

Meteorological factors and air pollutants were modelled simultaneously via the cross basis function in DLNM to account for the potential lagged and nonlinear effects^10^. Following many existing studies^18^ and trials and errors, the long-term time trend (DOS) was modelled by natural cubic splines with DOF of 7 per year (7*3 = 21 in total), while Month, DOW and Holiday were adjusted as categorical factors. Square root transformation was performed on wind, air pressure, CO and O3, and natural logarithmic transformation $log1p = {\text{log}}\left( {1 + x} \right)$ was performed on PM2.5, SO2, and NO2 to reduce their skewness. In cross basis function, a DOF of 2 or 3 were used to describe the nonlinear relationship, and 2 or 3 knots (3 or 4 DOF equivalently for natural cubic spline) were equally defined in the log spaced range. Following the existing studies that a relatively longer lagged effect was observed for meteorological factors than air pollutants^10^, a maximum lag of 26–34 days and 6–12 days were chosen for meteorological factors and air pollutants. These 6 parameters were optimized by the qAIC value^20^.

Considering the underlying nonlinear associations between factors and response in DLNM, the reference values for comparison depended on the shape (e.g. monotonic association, U-shaped association, etc.) of the identified associations^10,^^20^. In this study, the value corresponding to the minimum/maximum risk of COPD hospitalizations was chosen, which for hockey stick-shaped, U-shaped or V-shaped associations (or their inverted versions) could be estimated by inspecting the cumulative RR plot and the relative risk fit in $dlnm()$^20^. The lagged effects of different factors were evaluated using plots of RRs and cumulative RRs against lag days. Particularly, the effective lagged days were defined by which the lagged effects persist significantly.

### Subgroup analysis

Subgroup analysis by sex and age was conducted to assess their modified effects on the associations so that more detailed observations could be drawn.

### Model diagnostics and sensitively analysis

Deviance residual analysis including plot against time, histogram, and autocorrelation function (ACF) was adopted to evaluate the validity of the models. The deviance residuals of an ideal model were small, Gaussian distributed and with most ACF values being within the 95% confidence interval. Sensitivity analysis was conducted to assess model robustness by choosing different maximum lag days, DOFs and knots of the cross-basis functions for both meteorological factors and air pollutants.

### Ethical considerations

All data were fully anonymized for research purpose and the retrieved data included daily hospitalization number, sex and age. Ethics approval has been granted by the Ethics Committee at the University of Macau (BSERE20-APP005-ICMS), with a waiver regarding informed consent. It was also confirmed that all data collection was performed in accordance with relevant guidelines and regulations.

## Supplementary Information

Supplementary Information.
