# Supplementary material for: The short-term associations of chronic obstructive pulmonary disease hospitalizations with meteorological factors and air pollutants in Southwest China: a time-series study
Source: Sci Rep. 2021 Jun 21;11:12914. doi: 10.1038/s41598-021-92380-z (PMC8217527; doi:10.1038/s41598-021-92380-z)
Supplement: Supplementary file 1 — Supplementary Information. [file 41598_2021_92380_MOESM1_ESM.docx]

**The short-term associations of chronic obstructive pulmonary disease hospitalizations with meteorological factors and air pollutants in Southwest China: a time-series study**

Meng Li^1,†^, Shengqi Chen^1,†^, Hanqing Zhao^2^, Chengxiang Tang^3^, Yunfeng Lai^1^, Carolina Oi Lam Ung^1^, Jinya Su^4,*^, Hao Hu^1,*^

^1^ State Key Laboratory in Quality Research of Chinese Medicine, Institute of Chinese Medical Sciences, University of Macau, Macao SAR, China.

^2^ Sichuan Academy of Medical Sciences, Sichuan Provincial People’s Hospital, Chengdu, China.

^3^ School of Public Administration, Guangzhou University, Guangzhou, China.

^4^ School of Computer Science and Electronic Engineering, University of Essex, Colchester, CO4 3SQ, U.K.

*Correspondences: Jinya Su ([j.su@essex.ac.uk](mailto:j.su@essex.ac.uk)); Hao Hu ([haohu@um.edu.mo](mailto:haohu@um.edu.mo))

^†^These authors contributed as co-first authorship to the paper.

|  | NoH | T | DT | RH | W | P | PM2.5 | PM10 | SO2 | CO | NO2 | O3 |
| --- | --- | --- | --- | --- | --- | --- | --- | --- | --- | --- | --- | --- |
| NoH | 1 |  |  |  |  |  |  |  |  |  |  |  |
| T | -0.54 | 1 |  |  |  |  |  |  |  |  |  |  |
| DT | -0.50 | 0.93 | 1 |  |  |  |  |  |  |  |  |  |
| RH | 0.10 | -0.13 | 0.21 | 1 |  |  |  |  |  |  |  |  |
| W | -0.19 | 0.23 | 0.17 | -0.18 | 1 |  |  |  |  |  |  |  |
| P | 0.46 | -0.85 | -0.79 | 0.12 | -0.23 | 1 |  |  |  |  |  |  |
| PM2.5 | 0.33 | -0.45 | -0.46 | -0.01* | -0.51 | 0.32 | 1 |  |  |  |  |  |
| PM10 | 0.32 | -0.41 | -0.46 | -0.10 | -0.49 | 0.29 | 0.97 | 1 |  |  |  |  |
| SO2 | 0.26 | -0.19 | -0.26 | -0.21 | -0.32 | 0.12 | 0.64 | 0.68 | 1 |  |  |  |
| CO | 0.33 | -0.43 | -0.37 | 0.15 | -0.53 | 0.30 | 0.79 | 0.75 | 0.58 | 1 |  |  |
| NO2 | 0.37 | -0.32 | -0.34 | -0.04* | -0.61 | 0.29 | 0.76 | 0.78 | 0.63 | 0.71 | 1 |  |
| O3 | -0.42 | 0.74 | 0.54 | -0.54 | 0.19 | -0.65 | -0.28 | -0.22 | -0.10 | -0.36 | -0.20 | 1 |

**Supplementary Table S1.** Spearman correlation coefficients for COPD hospitalizations, meteorological factors and air pollutants, where the ones with * implies insignificant at the level of *P*<0.01.


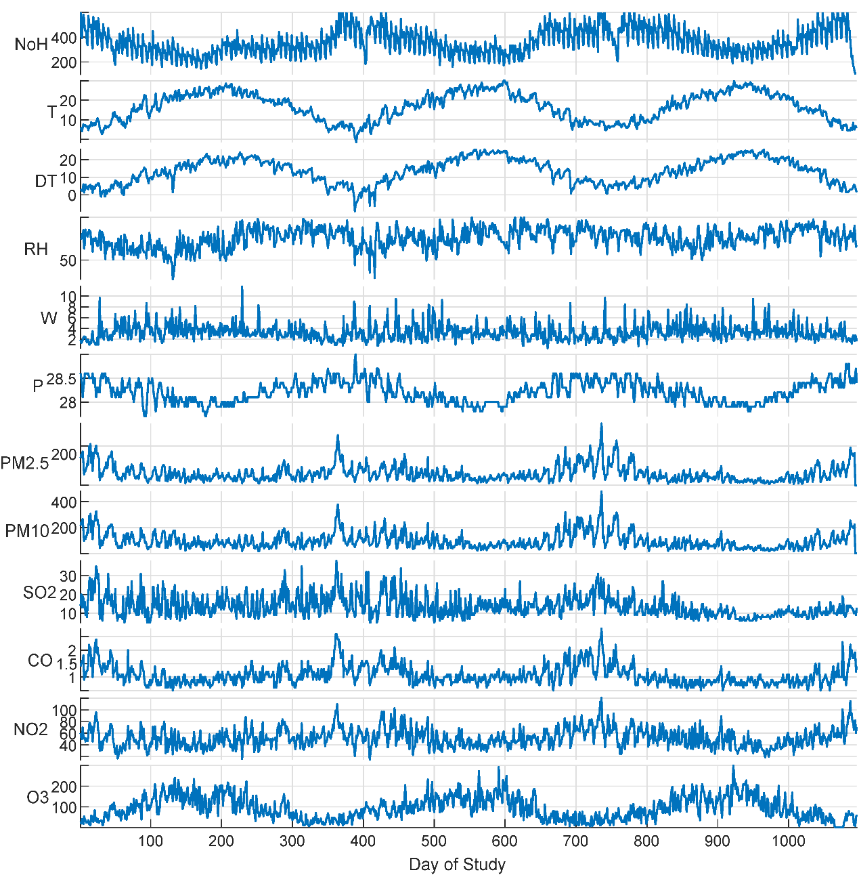


**Supplementary Figure S1.** Stacked plot of COPD hospitalizations against meteorological factors and air pollutants in Chengdu, China during Jan/2015-Dec/2017.
